# Carney complex (CNC)

**DOI:** 10.1186/1750-1172-1-21

**Published:** 2006-06-06

**Authors:** Jérôme Bertherat

**Affiliations:** 1 Centre de Référence Maladies Rares de la Surrénale, Service d'Endocrinologie, Hôpital Cochin, INSERM U 567, CNRS UMR 8104, Institut Cochin, Université René-Descartes Paris 5, Paris, 75014, France

## Abstract

The Carney complex (CNC) is a dominantly inherited syndrome characterized by spotty skin pigmentation, endocrine overactivity and myxomas. Skin pigmentation anomalies include lentigines and blue naevi. The most common endocrine gland manifestations are acromegaly, thyroid and testicular tumors, and adrenocorticotropic hormone (ACTH)-independent Cushing's syndrome due to primary pigmented nodular adrenocortical disease (PPNAD). PPNAD, a rare cause of Cushing's syndrome, is due to primary bilateral adrenal defect that can be also observed in some patients without other CNC manifestations or familial history of the disease. Myxomas can be observed in the heart, skin and breast. Cardiac myxomas can develop in any cardiac chamber and may be multiple. One of the putative CNC genes located on 17q22-24, (*PRKAR1A*), has been identified to encode the regulatory subunit (R1A) of protein kinase A. Heterozygous inactivating mutations of *PRKAR1A *were reported initially in 45 to 65 % of CNC index cases, and may be present in about 80 % of the CNC families presenting mainly with Cushing's syndrome. PRKAR1A is a key component of the cAMP signaling pathway that has been implicated in endocrine tumorigenesis and could, at least partly, function as a tumor suppressor gene. Genetic analysis should be proposed to all CNC index cases. Patients with CNC or with a genetic predisposition to CNC should have regular screening for manifestations of the disease. Clinical work-up for all the manifestations of CNC should be performed at least once a year in all patients and should start in infancy. Cardiac myxomas require surgical removal. Treatment of the other manifestations of CNC should be discussed and may include follow-up, surgery, or medical treatment depending on the location of the tumor, its size, the existence of clinical signs of tumor mass or hormonal excess, and the suspicion of malignancy. Bilateral adrenalectomy is the most common treatment for Cushing's syndrome due to PPNAD.

## Disease name/synonyms

Carney complex.

The complex of cardiac myxomas, endocrine overactivity and spotty pigmentation.

## Definition/diagnostic criteria

The Carney complex (CNC) was first described in 1985 by J. Aidan Carney, as the combination of myxomas, spotty pigmentation and endocrine overactivity [1]. It is defined by the association of multiple endocrine neoplasia and cardiocutaneous manifestations. Patients previously characterized as LAMB (lentigineses, atrial myxoma, mucocutaneous myxoma, blue nevi) or NAME (nevi, atrial myxoma, myxoid neurofibroma, ephelide) could be considered as having Carney complex. Numerous organs may be involved in CNC and the manifestations vary greatly among patients. Some of them are quite specific, such as primary pigmented nodular disease (PPNAD), while others show little specificity, such as thyroid nodes or blue nevi. It is generally assumed that a patient presenting with two or more of the manifestations listed in Table 1 would be diagnosed as having Carney complex. This table lists the most frequent features of CNC and their estimated frequency [according to references [1,3-5,8] and our personal observations]. The incidence of each manifestation depends on its presentation and might not reflect true prevalence. For instance, according to autopsy studies PPNAD is a constant feature in CNC patients [8], however, reports of Cushing syndrome in the literature indicate that only 25 to 45 % of CNC patients have PPNAD. It has been established that at least two of these manifestations need to be present to confirm the diagnosis of CNC. If the patient has a germline *PRKAR1A *mutation and/or a first-degree relative affected by CNC, a single manifestation is sufficient for the diagnosis.

**Table 1:** Main features and diagnostic criteria of Carney complex. This table lists the most frequent features of CNC and their estimated frequency [according to references 1, 3–5, 8 and our personal observations]. The incidence of each manifestation depends on its presentation and might not reflect true prevalence. For instance, according to autopsy studies PPNAD is a constant feature in CNC patients [8], however, reports of Cushing syndrome in the literature indicate that only 25 to 45 % of CNC patients have PPNAD and our personnal experience suggests that about 60 % of CNC patients systematically investigated have PPNAD. It has been established that at least two of these manifestations (except ovrian cyst) need to be present to confirm the diagnosis of CNC. If the patient has a germline *PRKAR1A *mutation and/or a first-degree relative affected by CNC, a single manifestation is sufficient for the diagnosis.

**Main features of Carney complex**	**(%)**
Primary Pigmented Nodular Adrenocortical Disease (PPNAD)	25–60
Cardiac myxoma	30–60
Skin myxoma	20–63
Lentiginosis	60–70
Multiple blue nevus	
Breast ductal adenoma	25
Testicular tumors (LCCSCT: Large-Cell Calcifying Sertoli Cell Tumor) (in male)	33–56
Ovarian cyst (in female)	20–67
Acromegaly	10
Thyroid tumor	10–25
Melanotic schwannoma	8–18
Osteochondromyxoma	<10

## Epidemiology

CNC is a rare disease. About 500 patients have been registered by the NIH-Mayo Clinic (USA) and the Cochin center (France) [2]. Cumulative reports from these centers, plus information from the Cornell center in New York, indicate that there are about 160 index cases of CNC presently known [3-6].

## Clinical description

The manifestations of CNC can be numerous and vary between patients. Even in the same kindred, phenotypic variability can be observed. The estimated frequencies of these manifestations are listed in Table 1. Endocrine, dermatologic and cardiac anomalies are the main manifestations of the disease.

### Skin lesions

The lentiginosis is observed in most patients and is so characteristic that can makes the diagnosis. It appears as small (2 to 10 mm) brown to black macules typically located around the upper and lower lips, on the eyelids, ears and the genital area. Multiple blue nevi and junctional or compound nevi may also be observed in CNC, as well as cutaneous myxomas. The skin myxomas present as non-pigmented subcutaneous nodules. Myxomas can also be located in the ear canal.

### Endocrine tumors

**Figure 1:**
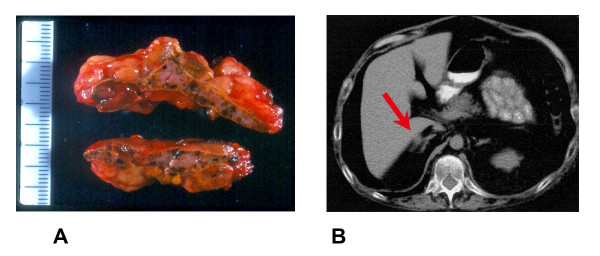
Macroscopic and CT-scan findings in primary pigmented nodular adrenocortical disease (PPNAD) A: Macroscopic appearance of the adrenal gland in PPNAD. The cut surfaces show multiple pigmented micronodules. The periadrenal fat is also visible around the adrenal capsule. B: Adrenal CT-scan in PPNAD. A micronodule is visible on the external part of the left adrenal on the CT-scan shown (see red arrow).

The following types of endocrine gland tumors are observed in CNC patients: growth hormone (GH)-secreting pituitary adenomas (acromegaly), thyroid adenomas or carcinomas, testicular tumors (large-cell calcifying Sertoli cell tumors), and ovarian cysts. Adrenocorticotropic hormone (ACTH)-independent Cushing's syndrome due to PPNAD is observed in 25 to 30 % of patients with CNC. PPNAD is a rare disease observed mostly in patients with CNC. The disease was named after the macroscopic appearance of the adrenals that is characterized by the small pigmented micronodules observed in the cortex (Figure 1) [7]. The disease is usually bilateral with primary involvement of both adrenals. Cushing's syndrome due to PPNAD is observed in children and young adults, with a peak during the second decade of life [8]. It is rare, but can occur before the age of 4-yr and it is rarely diagnosed after the age of 40-yr. Diagnosis of Cushing's syndrome due to PPNAD is often difficult because hypercortisolism can develop progressively over years. In contrast, a large and rapid burst of cortisol excess can be observed in some patients, which might spontaneously regress. In some cases of PPNAD, clearly cyclic forms of hypercortisolism have been documented [6,9,10]. PPNAD can also be diagnosed by systematic screening in patients with CNC investigated for other clinical manifestations of the complex or after familial screening. Despite the unusual time course of Cushing's syndrome observed in some patients with PPNAD, clinical signs are quite similar to those observed in patients presenting with other causes of hypercortisolism. Urinary cortisol is increased in most patients at the time of diagnosis of PPNAD, but its level can be highly variable. The circadian rhythm of cortisol secretion is usually completely abolished. As with ACTH-independent Cushing's syndrome due to other causes, patients with PPNAD have low plasma levels of ACTH and show no stimulation of cortisol or ACTH secretion after corticotropin-releasing hormone (CRH) injection. In addition, dexamethasone fails to suppress cortisol secretion, even after high dose administration. Pathological investigation reveals that adrenal glands from patients with PPNAD are usually normal in size and weight (between 4 to 17 g) [8]. In keeping with this finding, adrenals appear normal on computed tomography (CT)-scan in one out of three patients (Figure 1). In the other patients, micronodules can be visible and, more rarely, macronodules (>1 cm diameter) in one or both glands. Iodocholestrol scintigraphy, when performed, usually shows a bilateral uptake despite ACTH suppression by endogenous hypercortisolism.

Acromegaly due to a pituitary GH-secreting tumor is not very frequent, but most patients with CNC present with a mild increase in GH, and sometimes in prolactin (PRL) secretion. Alterations in the rhythm of GH secretion are frequently observed.

Thyroid tumors are most often benign, non-toxic adenomas, mostly of follicular type. Some patients present with papillary carcinoma that can be multiple and sometimes quite aggressive.

Testicular tumors are easily detected by ultrasound investigation as bilateral microcalcifications. Ovarian cysts and cystadenoma have been observed in CNC patients.

### Cardiac myxomas

Cardiac myxoma is an important manifestation of CNC. It may be the cause of the high rate (16 %) of sudden death historically reported in CNC families [11], thus underlying the importance of its early diagnosis. In the past, underdiagnosis of cardiac myxomas may have accounted for the majority of deaths due to CNC. In contrast with sporadic myxoma, they can develop in any cardiac chamber and may be multiple. Cardiac myxoma can be the cause of stroke due to embolism and cardiac deficiency. It is therefore important to screen regularly (by ultrasound) patients with CNC for the presence of cardiac myxoma. In difficult cases, trans-esophageal ultrasound and cardiac magnetic resonance imaging (MRI) can be very helpful.

### Other tumors

Various other tumors, some of them quite specific for CNC, can be observed. Melanotic schwannoma is a rare tumor and occurs mainly in CNC. It is a pigmented tumor that can be misdiagnosed as a melanoma. This tumor can be observed in any periphal nerve and can be, in rare cases, malignant. Breast ductal adenomas, breast myxomas, and osteochondromyxoma are among the tumors also observed in CNC.

## Etiology/genetics of CNC

The first description of CNC included 40 patients [1], among them 10 were familial cases, leading to the hypothesis of a genetic origin, at least in a subset of patients. CNC seems to be a genetically heterogeneous disease and linkage analysis has shown that at least two loci are involved: 2p16 and 17q22-24 [11,12]. The *CNC1 *gene, located on 17q22-24, has been identified as the regulatory subunit (R1A) of the protein kinase A (*PRKAR1A*) [13,14]. PRKAR1A is a key component of the cAMP signaling pathway that has been implicated in endocrine tumorigenesis. Heterozygous inactivating mutations of *PRKAR1A *have been detected in about 45 to 65 % of CNC families [3,4]. In CNC patients with Cushing's syndrome the frequency of *PRKAR1A *mutations is about 80 %, suggesting that families with PPNAD are more likely to carry a 17q22-24 defect [5]. Interestingly, patients with isolated PPNAD and no familial history of CNC may also carry a germline *de novo *mutation in *PRKAR1A *[6]. In the tumors of CNC patients loss of heterozygosity (LOH) at 17q22-24 may be observed, suggesting that *PRKAR1A *is a tumor suppressor gene. Somatic mutation of *PRKAR1A *in a patient with PPNAD already carrying a germline mutation may lead to inactivation of the wild type allele [6]. However, inactivation of the remaining wild type allele by genetic alteration does not appear to be a constant step in PPNAD and CNC tumor development [5]. In a mice transgenic model with heterozygous inactivation of *PRKAR1A*, tumors may develop without allelic loss [4]. This suggests that the classic model of tumor suppressor gene with a germline inactivating first allelic alteration, followed by a second genetic hit leading to inactivation of the remaining wild type allele, might to some extent be applicable to PRKAR1A. It is also possible that in PPNAD, a general polyclonal expansion might be stimulated by haploinsufficiency due to the first germline defect; a second genetic hit would then lead to inactivation of the wild type allele and further stimulate tumorigenesis and the development of adrenocortical nodules.

A putative *CNC2 *gene located at the 2p16 locus remains to be determined [3,11]. Somatic alterations of the 2p16 region have been reported in CNC tumors, even in patients with mutation of the *CNC1 *gene (*i.e. PRKAR1A *located on 17q22-24) [15]. These alterations are usually gene amplifications, suggesting that the gene located at 2p16 is a potential oncogene. Considering the genetics in isolated PPNAD, it should be mentioned that the clinical manifestations in a subgroup of very young PPNAD patients may differ from those in older patients with CNC. In these patients the classical pathological finding of pigmented nodules may be absent although micronodules are present [10,16]. In this subgroup of very young PPNAD patients, Cushing's syndrome may occur between birth and the age of 5-yrs. The main reason for differentiating this group of PPNAD or PPNAD-like patients is the lower rate of germline inactivating mutation.

## Management including treatment

Patients with CNC or with a genetic predisposition to CNC (*i.e. PRKAR1A *germline mutation carriers) should have regular screening for manifestations of the disease. At present, it is not possible to produce evidenced-based recommendations for the screening schedule. However, there is general agreement on the following recommendations. Clinical work-up for all the manifestations of CNC should be performed at least once a year in all patients and should start in infancy. Cardiac myxomas are one of the main features of CNC and should be diagnosed early through screening performed at least once a year by cardiac ultrasound. In patients with a history of cardiac myxoma, screening should be performed every 6 months. Screening for cardiac myxoma by ultrasound should start during the first 6 months. In contrast, screening for the other manifestations (only by clinical examination) should be performed in patients under 5 years-old. For children, pubertal staging and growth rate should be monitored. Biological and hormonal work-up should include: measurement of the levels of blood glucose, urinary cortisol, plasma and/or salivary circadian variations of cortisol, plasma ACTH, GH, PRL, Insulin-Like-Growth-Factor I, and ionograms. Imaging investigations should include: adrenal CT-scan (in cases with biological evidence of Cushing's syndrome), thyroid ultrasound (in cases with abnormal palpation), testicular or ovarian ultrasound, pituitary MRI, and spine MRI when the clinical signs suggest schwannoma. These imaging investigations do not always need to be performed each year; the necessity will be determined according to the history and previous imaging results, as well as the present clinical data and results of biological investigations.

A cardiac myxoma requires surgical removal. Treatment of the other manifestations should be discussed and may include follow-up, surgery, or medical treatment depending on the location of the tumor, its size, the existence of clinical signs of tumor mass or hormonal excess, and the suspicion of malignancy. Malignancy in CNC is mostly observed in thyroid nodes (fine-needle investigation is helpful for diagnosis) and melanotic schwannoma (estimated rate of malignancy: 10 %). Bilateral adrenalectomy is the most common treatment for Cushing's syndrome due to PPNAD. Some rare cases have been treated by O, p'-dichlorodiphenyldichloroethane (DDD), ketoconazol, or unilateral adrenalectomy. In the few patients in whom overt Cushing's syndrome did not recur after unilateral adrenalectomy, alterations in the rhythm of cortisol secretion can be observed on long term follow-up, demonstrating that despite apparent cure the disease is indeed bilateral [17].

## Genetic counseling

Genetic analysis should be proposed to all CNC index cases. When a *PRKAR1A *mutation is identified, a genetic analysis should be proposed to first degree relatives. This is best performed in a multidisciplinary genetic center, in keeping with the regulations specific to each country. Psychological assessment and management of the patients might be important when performing genetic screening in asymptomatic patients. The identification of mutation carriers should lead to the same follow-up and management as that described for CNC patients. Therefore, it is suggested that genetic screening of first degree relatives is performed at the same time as the first cardiac ultrasound. When performing genetic screening in an asymptomatic child, the parent should be given information about the fact that a positive screening with identification of a *PRKAR1A *mutation should lead to regular screening and follow-up for CNC manifestations. At present, no recommendation has been made for prenatal diagnosis and this should be discussed by a multidisciplinary team in keeping with the regulation specific to each country. There are no reports of prenatal diagnosis of CNC in Europe.

## Unresolved questions

No *PRKAR1A *gene mutations were found in some families and the 17q22-24 locus was excluded. The other gene(s) responsible for CNC remain to be identified. In CNC kindred without the *PRKAR1A *mutation, a large gene deletion or alteration (not detected by the commonly used direct sequencing or DHPLC methods) may be responsible for the condition.

A mutation of the *perinatal myosin heavy chain *gene has been reported in patients with trismus-pseudocamptodactyly syndrome. In some of the cases, manifestations of this syndrome overlap with those observed in CNC [18]. However, the phenotype differs from CNC and at present it does not seem that this gene is involved in kindreds with the classic diagnostic criteria of CNC [19].

The subgroup of very young infants with isolated PPNAD, no other personal or familial history of CNC and no *PRKAR1A *mutation may have a separate disease.

Some rare manifestations of the disease (such as osteochondromyxoma) have not yet been extensively described.

The mechanism of *PRKAR1A *mutation-induced tumorigenesis is currently under extensive investigation and is an important field of research.
